# *Culicoides* Biting Midges—Underestimated Vectors for Arboviruses of Public Health and Veterinary Importance

**DOI:** 10.3390/v11040376

**Published:** 2019-04-24

**Authors:** Franziska Sick, Martin Beer, Helge Kampen, Kerstin Wernike

**Affiliations:** 1 Institute of Diagnostic Virology, Friedrich-Loeffler-Institut, Suedufer 10, 17493 Greifswald-Insel Riems, Germany; Franziska.Sick@fli.de (F.S.); Martin.Beer@fli.de (M.B.); 2 Institute of Infectology, Friedrich-Loeffler-Institut, Suedufer 10, 17493 Greifswald-Insel Riems, Germany; Helge.Kampen@fli.de

**Keywords:** *Culicoides*, biting midges, transmission, insect vector, *Bunyavirales*, orthobunyavirus, Simbu serogroup, Schmallenberg virus, Akabane virus

## Abstract

*Culicoides* biting midges, small hematophagous dipterans, are the demonstrated or putative vectors of multiple arboviruses of veterinary and public health importance. Despite its relevance in disease spread, the ceratopogonid genus *Culicoides* is still a largely neglected group of species, predominantly because the major human-affecting arboviruses are considered to be transmitted by mosquitoes. However, when a pathogen is detected in a certain vector species, a thorough search for further vectors often remains undone and, therefore, the relevant vector species may remain unknown. Furthermore, for many hematophagous arthropods, true vector competence is often merely suspected and not experimentally proven. Therefore, we aim to illuminate the general impact of *Culicoides* biting midges and to summarize the knowledge about biting midge-borne disease agents using the order *Bunyavirales*, the largest and most diverse group of RNA viruses, as an example. When considering only viruses evidentially transmitted by *Culicoides* midges, the Simbu serogroup (genus *Orthobunyavirus*) is presumably the most important group within the virus order. Its members are of great veterinary importance, as a variety of simbuviruses, e.g., the species *Akabane orthobunyavirus* or *Schmallenberg orthobunyavirus*, induces severe congenital infections in pregnant animals. The major zoonotic representative of this serogroup occurs in South and Central America and causes the so-called Oropouche fever, an acute febrile illness in humans.

## 1. Introduction

During the last decade, the incidence of emerging viral diseases has increased considerably in various regions worldwide. Diseases such as Zika fever or West Nile fever have been among numerous neglected viral diseases, until they have expanded to new regions and caused large outbreaks, thereby attracting strong media attention [1,2]. Among the emerging or re-emerging pathogenic viruses are a large number of so-called arthropod-borne (arbo) viruses, i.e., viruses that are transmitted between their vertebrate hosts by insects and other arthropods [3].

For the establishment and maintenance of “virus-vector-host” transmission cycles of arboviruses in a given area, competent vectors and susceptible hosts need to encounter under favorable environmental conditions [4]. Hence, as reasons for the increasing number of reported arbovirus-induced disease outbreaks, factors such as climate change, increasing urbanization, and globalization with increasing trade, livestock movement, and increasing traveling activities are discussed [5].

Medically, one of the most highly important arthropod vectors is unquestionably mosquitoes. Nonetheless, further, sometimes neglected arthropods such as ticks or biting midges can also act as vectors of emerging disease agents [6]. However, when a pathogen has been detected in a certain vector species, a thorough search for further putative vectors often remains undone. Furthermore, the mere demonstration of a pathogen in a blood-feeding arthropod is often mistaken as evidence for vector competence of that arthropod species without further experimental studies. Therefore, we want to summarize here knowledge about ceratopogonid-transmitted disease agents and their impact on human and animal health focusing on *Culicoides* biting midges and the new viral order *Bunyavirales*, which contains the largest and most diverse families of RNA viruses [7]. In this review, we illuminate the general impact of *Culicoides* biting midges, an often-neglected insect vector, but primarily, we present evidence of their role in the transmission of selected orthobunyaviruses of public health or veterinary importance.

## 2. How to Differentiate Mechanical from Biological Vectors?

Vector competence refers to the physiological ability of arthropods to acquire and maintain microbial agents from a host, and later transmit them to the next susceptible host [8]. The mere virus detection in a midge does not necessarily indicate vector competence. A midge feeding on a viremic host can carry virus from the blood meal in its gut without being infected itself. However, it can subsequently serve as a so-called “mechanical vector” for virus transmission. To understand a disease and its epidemiology entirely, it is essential to distinguish biological (competent) from pure mechanical vectors.

One option to examine the vector competence is the separate analysis of the head and the salivary glands and the rest of the insect body. Virus detection in the salivary glands is a sign for a biological infection followed by virus dissemination, resulting in a potential virus transmission via saliva. Hence, virus detection in the salivary glands of an insect hints at true vector competence. On the other hand, detection of virus in the completely homogenized body of the insect may indicate just the ingestion of a viremic blood meal, which not necessarily leads to an infection of the insect.

To evaluate vector competence in more detail, infection studies under laboratory conditions either with biting midges caught in the field or with laboratory-adapted colonies are a suitable method. Insects can be artificially infected by intrathoracic inoculation of virus directly into the hemolymph bypassing the intestinal (“gut”) barrier, or orally by feeding viremic blood. Subsequently, after an extrinsic incubation period, samples are collected from surviving individuals. The insects themselves could be examined as a whole or with head plus salivary glands and body separated as described above. Furthermore, saliva could be collected from individual insects and tested for infectious virus or viral genome. From the results, infection, dissemination and transmission rates are calculable [9].

## 3. *Culicoides* Biting Midges: Classification, Morphological Characteristics, and Distribution

*Culicoides* is a genus of biting midges in the order Diptera, family Ceratopogonidae. Currently, the genus contains 1368 species divided into numerous subgenera [10].

*Culicoides* biting midges are among the most abundant hematophagous insects worldwide, occurring from temperate areas to the tropics. Representing one of the smallest hematophagous flies, they measure only 1–3 mm [11]. Their mouthparts form a proboscis well adapted for cutting skin and sucking blood. Wings are well developed, and biting midges are commonly identified to complex or species level based on the wing maculation [12]. However, this method of identification is very time consuming and depends on the professional experience of the examiner. Alternative methods use genome amplification by polymerase chain reaction (PCR) with subsequent sequencing and phylogenetic comparison of DNA marker regions [13,14]. In addition, real-time PCR assays have been established for a few *Culicoides* species [15,16], a DNA microarray was developed to identify *Culicoides* species of the obsoletus group [17], and matrix-assisted laser desorption/ionization time-of flight mass spectrometry (MALDI-TOF-MS) has been used to identify *Culicoides* species based on peptide and protein signatures [18].

The life cycle of *Culicoides*, which passes through the egg stage, four larval stages, a pupa, and the imago, requires a certain amount of free water or moisture, and some species occur in both fresh water and estuarine environments. Breeding sites range from pools, streams, tree holes to saturated soil, animal dung and rotting vegetation [19]. Females depend on blood for the maturation of the eggs; they feed, depending on the species, on mammals and/or birds. Males do not feed on blood, and can survive on nectar alone. The development of *Culicoides* species takes a few weeks—or even months, when overwintering in a larval stage—and this process depends on the ambient temperatures, resulting in a seasonal activity pattern in temperate regions. In mild climatic zones, the insect numbers start to increase in late spring and early summer and usually peak in late summer or early autumn [11,20]. With the onset of low temperatures and the first frosts, the number of active insects drops dramatically.

In general, adult biting midges are short-lived and only a few individuals survive longer than 10 to 20 days. During this time, females may feed on hosts multiple times [11].

## 4. Public Health and Veterinary Impact of *Culicoides* Biting Midges

Biting midge bites can cause painful lesions; in some cases, the saliva even induces acute allergic reactions such as the “common summer eczema (insect hypersensitivity)” in horses [21]. However, their veterinary or public health importance predominantly results from their role in the transmission of pathogens, especially viruses, but also protozoans and filarial parasites [22] such as avian hamosporidians [23,24] and *Tetrapetalonema* spp. [25,26,27]. More than 50 viruses have been isolated from *Culicoides* spp. worldwide [28,29]. Most of the viruses belong to the families *Reoviridae* (e.g., African horse sickness virus (AHSV), bluetongue virus (BTV), or epizootic hemorrhagic disease virus (EHDV)), *Rhabdoviridae* (e.g., bovine ephemeral fever virus (BEFV)) and *Peribunyaviridae* (e.g., Akabane virus (AKAV), Schmallenberg virus (SBV), or Oropouche virus (OROV)). However, some viruses isolated from *Culicoides* are only incidental findings, such as Rift Valley fever virus (RVFV), which is mainly transmitted by mosquitoes [28,30]. Furthermore, vector competence studies are still missing for a wide range of viruses suspected to be transmitted by *Culicoides*.

Surveillance on biting midges and biting midge-borne diseases is carried out in various countries of several continents. As an example, the distribution and potential spread of BTV is monitored by the collection of biting midges in virtually every affected European country, also to define e.g., a so-called “seasonally vector free” period. To sample the adult *Culicoides* population, light-suction traps placed at sentinel sites across outbreak areas are the most commonly used standardized trap system [31]. Furthermore, detailed studies on local dispersal of *Culicoides* are available from Australia, comparing various types of light traps with different outcomes in biting midge catches [32] and analyzing distribution patterns related to weather and climate [33].

In search for potential vector species, an accurate estimate of species actually biting the vertebrate host is vital. In the context of BTV vector search in Europe, it was demonstrated in a comparative study of light-suction traps and drop catches that the biting midges caught in light-suction traps do not provide an accurate reflection of the biting *Culicoides* population [31], which has to be considered when assessing data collected in such surveillance studies.

Because of the needs of their agents’ vectors, *Culicoides*-borne diseases are strongly linked to climate and weather. In temperate regions, the seasonal pattern of virus transmission coincides with warm, moist summer and autumn months. In tropical and subtropical regions, high vertebrate infection rates have been reported during wet summer and declining transmission rates in periods of lower rainfall [34]. Although their flight range does not exceed a few hundred meters, biting midges can be dispersed passively over great distances by wind [35,36,37,38], further contributing to their impact on disease epidemiology.

The establishment of laboratory colonies poses great difficulties, especially because field-collected specimens fail to mate under laboratory conditions. So far, very few *Culicoides* species have been successfully reared under laboratory conditions [29,39,40] and, consequently, are available for studies on their vector competence. Most laboratory colonies represent *C. nubeculosus* and *C. sonorensis*, rendering these two species important models for studying arbovirus transmission by *Culicoides* biting midges [41]. Due to difficulties in acquiring biting midges from the field for laboratory studies caused by the characteristics of the insects, the major part of data available on experimental infections pertain to *C. nubeculosus* and *C. sonorensis*.

## 5. The Genus Orthobunyavirus

Only recently, the International Committee on Taxonomy of Viruses (ICTV) made some fundamental changes in the taxonomy of the order *Bunyavirales*, which had been established in 2017 to accommodate viruses with linear, segmented, single-stranded RNA genome [42]. In early 2019, the order was taxonomically revised by creating several new families, subfamilies, genera, and species. As of February 2019, the order *Bunyavirales* consists of 46 genera assigned to 12 distinct families [7]. One of the genera belonging to the newly established family *Peribunyaviridae* is the genus *Orthobunyavirus*, which contains more than 170 unanimous insect-transmitted viruses in 18 serogroups, of which the Simbu serogroup is not only one of the largest, but also the most important in terms of veterinary public health. For human health, the California encephalitis serogroup is very likely the most relevant one as it contains, e.g., La Crosse virus (LACV).

In general, orthobunyaviruses are spherical, about 100 nm in diameter and relatively simple in their composition as the tri-partite RNA genome encodes only four structural and two non-structural proteins. The small (S) genomic segment encodes for the nucleocapsid protein N and the non-structural protein NSs, while the medium (M) segment encodes for the glycoproteins Gn and Gc, which form spikes on the surface of the virus particle, and the non-structural protein NSm. The large (L) segment encodes for the RNA dependent RNA polymerase [43].

The glycoproteins Gn and Gc are integral transmembrane proteins which are important for viral attachment, membrane fusion and the induction of the host’s immune response [44,45,46]. The N-protein is essential for viral RNA transcription and replication, and it forms ribonucleoprotein complexes with the three viral RNA segments. These complexes are associated with the L-protein [47,48]. The NSs-protein is a major virulence factor in vertebrate hosts as it acts as an interferon antagonist and is responsible for the shut-off of protein synthesis in mammalian hosts cells [48]. In vertebrate hosts, the NSm-protein is associated with virus assembly and morphogenesis [49]. However, the function of both non-structural proteins, NSs and NSm, in the insect vectors is largely unknown.

### 5.1. The Simbu Serogroup

**Figure 1:**
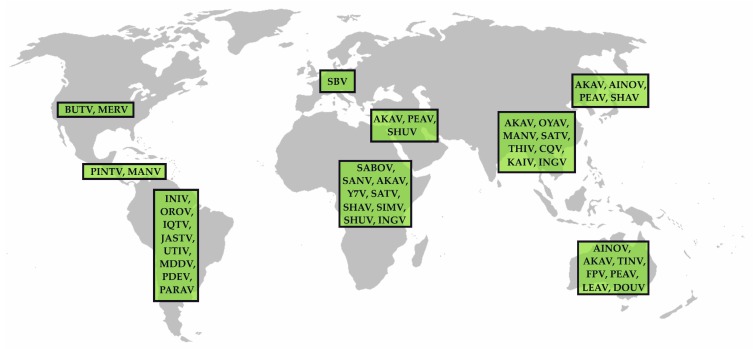
Distribution of Simbu serogroup viruses. The meanings of the abbreviations (virus names) are listed in Table 1.

When considering only viruses evidentially transmitted by *Culicoides* midges, the Simbu serogroup is presumably the most important group within the genus *Orthobunyavirus*. Furthermore, its members are of great veterinary importance, as a variety of simbuviruses induces severe congenital infections in pregnant animals. The serogroup currently consists of 32 viruses grouped into 19 virus species (Table 1). Simbuviruses are distributed worldwide (Table 1, Figure 1), and they persist in nature by alternately infecting mammalian hosts and *Culicoides* vectors. In endemic regions, Simbu serogroup viruses establish a pattern of cyclic circulation, with seasons of high virus appearance followed by periods of only sporadic detection [50,51,52,53,54,55], which is presumably related to the overall immunity in the host population and the abundance of *Culicoides* vectors.

Important representatives of the Simbu serogroup are AKAV and SBV, which predominantly infect ruminants. Infections of adults are either asymptomatic or mild, associated with fever, diarrhea, and decrease in milk yield for a few days. Infections of naïve dams during a critical phase of gestation, however, may be followed by abortion, premature birth, mummification, stillbirth or congenital deformations referred to as arthrogryposis-hydranencephaly syndrome [34,56]. Further ruminant-infecting Simbu serogroup viruses that might induce similar clinical signs as AKAV or SBV include Shuni virus (SHUV), Aino virus (AINOV) and the eponymous Simbu virus (SIMV) [57] (Table 1). The only zoonotic virus of this serogroup is OROV (and reassortants such as Iquitos virus and Madre de Dios virus), which is present in South America (Figure 1).

#### 5.1.1. AKAV

AKAV was first described in the 1950s in Japan and is now endemic in large parts of Asia, the Middle East, Australia and Africa [34,57,85,86]. The virus may induce abnormal courses of pregnancy and fetal malformation in ruminants as described for both AKAV and SBV [34]. In addition, some strains might occasionally cause encephalitis in newborn calves. The Iriki strain, which is present in Japan and Korea, has been in rare cases associated with encephalitis in adult cattle [87,88,89].

Although AKAV was initially isolated from mosquitoes, they do not seem to play an important epidemiological role in virus transmission [90]. In contrast, depending on geographical regions, various *Culicoides* species are considered the main vectors and responsible for virus spread.

For Australia, the assumed main vector is *C. brevitarsis* [91,92], since it was demonstrated that AKAV replicates in *C. brevitarsis* to high virus titers and reaches the salivary glands after 10 days of incubation [93]. Furthermore, the virus has been isolated from *C. wadai* in Australia [94]. In Japan, *C. oxystoma* is considered the major vector [51,95], although AKAV was also isolated from the mosquito species *Aedes vexans* and *Culex tritaeniorhynchus* [58], which are most likely no competent vectors. In the Middle East, *C. imicola* is probably the main vector responsible for virus circulation. In Israel, AKAV was repeatedly detected in this vector species by RT-PCR, and in Oman, the isolation of AKAV was possible from this culicoid species [96,97]. Also, throughout Africa, *C. imicola* seems to be an important vector of AKAV. The virus was isolated multiple times from *C. imicola* in Zimbabwe [98], and in South Africa AKAV was detected in a pool of mixed *Culicoides* spp. mainly consisting of *C. imicola* [99]. Another vector could be *C. milnei*, as AKAV was isolated from this species in Zimbabwe [98]. In Kenia, AKAV was isolated from *Anopheles funestus* mosquitoes [100], but true vector competence has not been demonstrated yet.

To investigate the vector competence of midges for AKAV, some experimental infection studies were carried out with different *Culicoides* species. It could be demonstrated that AKAV replicates in *C. variipennis* after oral infection for at least 9 days, while in *C. nubeculosus*, AKAV replicated only after intrathoracic inoculation [101]. The extensive replication of AKAV within *C. variipennis* suggests that *Culicoides* spp. can act as fully competent vectors. However, although *C. variipennis* serving as a suitable surrogate model, this laboratory-adapted species is not the natural vector of the virus, as it is native to North America while AKAV is endemic in Asia, Africa, and Australia.

#### 5.1.2. SBV

SBV emerged in late 2011 in the German/Dutch border region [79] and is now endemic in most European countries [50,102]. It predominantly infects ruminants, causing a mild, transient disease in adult animals, but it may induce severe fetal malformation when naïve dams are infected during a critical period of pregnancy [103]. Based on its close relationship to AKAV, it was assumed that *Culicoides* act as vectors also for SBV and, indeed, SBV genome was subsequently detected in field-collected *Culicoides* midges of various species repeatedly throughout Europe [104,105,106,107,108]. In contrast, mosquitoes do not seem to play a major role, if any, in virus transmission [109,110,111,112].

In temperate European countries, biting midges of the *C. obsoletus* complex are considered the main vectors, while in Mediterranean countries *C. imicola* and *C. punctatus* seem to significantly contribute to virus transmission. Viral genome was also detected in *C. dewulfi, C. pulicaris, C. newsteadi, C. lupicaris,* and *C. nubeculosus* by (real-time) RT-PCR [104,105,106,107,108,112,113,114]. To underpin the findings in field-collected biting midges with experimental data, several vector competence studies were carried out. The Nearctic species *C. sonorensis* was infected orally and intrathoracically. The detection of viral genome after an extrinsic incubation period in the saliva and the isolation of infectious virus from the head proved dissemination of SBV within the insect organism [115] and demonstrated that *C. sonorensis* is a suitable laboratory model for SBV. Furthermore, from these infected individuals, a range of Cq-values from decapitated heads (including salivary glands) was available to compare with those produced from heads of field-collected *Culicoides*. The values provided for field-collected *C. obsoletus, C. scoticus* and *C. chiopterus* from the Netherlands were very similar to those obtained from the laboratory infections, indicating that these species act as true vectors [115]. In contrast, multiple infection studies with laboratory colonies of *C. nubeculosus* showed only low infection rates, although viral genome was detected in field-collected *C. nubeculosus* [112,115].

Unfortunately, for the *C. obsoletus* complex, which consists of the species *C. obsoletus*, *C. scoticus*, *C. chiopterus* and *C. montanus* and is considered to contain the major vectors of SBV in temperate European countries, laboratory colonies are not available. However, experimental laboratory infection studies were carried out with field-collected individuals of the *C. obsoletus* complex and with *C. imicola*, which, according to field data, is considered the main vector in the Mediterranean. Both, i.e., midges of the *C. obsoletus* complex and *C. imicola*, showed high infection rates and virus dissemination [116], confirming the assumptions of their vector competence, and field-collected *C. scoticus* were able to replicate SBV to a potentially transmissible level [112].

#### 5.1.3. SHUV

SHUV was firstly isolated in Nigeria from healthy cattle [60] as well as from *Culicoides* biting midges and mosquitoes in Africa [117]. In 2014, SHUV was for the first time detected outside of Africa, and was isolated from malformed lambs in Israel [118]. In addition to typical Simbu virus-related congenital defects in cattle and sheep, SHUV may occasionally induce severe neurological symptoms in horses or cattle [119,120]. A general zoonotic potential cannot be ruled out, since SHUV was isolated from a febrile child in Nigeria [121], and specific antibodies were found in large animal veterinarians in South Africa [122].

In South Africa, SHUV was recovered twice from pools of *Culex theileri* [117]. However, the mosquito taxa *Culex pipiens* biotype *pipiens* and *Aedes aegypti* showed only very low susceptibility to SHUV following intrathoracic inoculation. Oral infection was not possible under experimental conditions [9]. In contrast, *C. nubeculosus* and *C. sonorensis* could be orally infected, with the virus disseminating well in both species [9], indicating that *Culicoides* biting midges are more likely the natural vectors than mosquitoes.

#### 5.1.4. OROV

First discovered in a febrile forest worker in 1955 in Trinidad [74], OROV is now endemic in many South and Central American countries [123,124]. Oropouche fever is an acute febrile illness that affects humans. Common symptoms include fever, headache, muscle pain and skin rash, and many infections develop into meningitis or encephalitis [123]. With this zoonotic potential, OROV protrudes from the other viruses in the Simbu serogroup. In South and Central America, OROV occurs in a sylvatic cycle between its insect vectors, some wild bird species and mammals such as rodents, sloths and non-human primates as amplifying hosts (Table 1). In the urban cycle, humans are most likely the only vertebrate hosts, as domestic animals such as chickens, dogs or cats could be excluded as amplifying hosts [124,125].

The main vector in the sylvatic cycle is still unclear; however, OROV was isolated from two sylvatic mosquitoes in the forest: *Aedes serratus*, collected in the Brazilian Amazon region, and *Coquillettidia venezuelensis* in Trinidad [125,126].

In the urban cycle, the biting midge species *C. paraensis* and the mosquito *Culex quinquefasciatus* are believed to be the main vectors. Although the isolation rate from *C. paraensis* during epidemics is low, its involvement as a vector is suggested based on transmission studies, where *C. paraensis* transmitted OROV to hamsters five or more days after feeding on blood of viremic patients [125].

Likewise, *Culex quinquefasciatus* proved to be an ineffective vector of OROV in laboratory transmission experiments [127], but virus is frequently detected in this species during outbreaks [128]. The link between the two transmission cycles, i.e., the sylvatic and the urban cycle, are probably humans entering the forests, where they become infected, and returning to urbanized areas [125].

#### 5.1.5. Further Members of the Simbu Serogroup

There are several additional members of veterinary importance within the Simbu serogroup (Table 1), which might induce the typical congenital malformation when naïve pregnant dams are infected. Unfortunately, vector competence studies are missing for most of them. However, in general, certain biting midge species are suspected to be able to transmit several members of the serogroup. As an example, field-collected *C. brevitarsis*, the major vector for AKAV in Australia, were tested positive for further Australian simbuviruses such as Douglas virus (DOUV) or Peaton virus (PEAV) [59,77]. In the Mediterranean and the Middle East, where *C. imicola* is the main vector of SBV or AKAV, also PEAV was found in this species [129], confirming the concept of certain *Culicoides* species transmitting several simbuviruses present in the respective region.

### 5.2. Further Orthobunyaviruses of Public Health Importance

In the California encephalitis serogroup of the genus *Orthobunyavirus*, several zoonotic agents distributed in North America, Europe, Africa, or Asia can be found. However, for none of them *Culicoides* spp. have been demonstrated to be vector-competent. Since they are transmitted by mosquitoes between their vertebrate hosts, which range from small mammals to ungulates and humans, they are among the viruses that are referred to as “mobovirus” (= mosquito-borne viruses) [130]. Some of the most relevant representatives of zoonotic orthobunyaviruses include LACV, Jamestown Canyon virus (JCV), Keystone virus (KEYV) and Ťahyňa virus (TAHV).

LACV, the most pathogenic agent within the California encephalitis serogroup, is the causative agent of the so-called “rural encephalitis” in the Appalachian and midwestern regions of North America and the leading cause of pediatric arboviral encephalitis. Clinical symptoms of an LACV-infection include headache, fever, myalgia, and encephalitis, while in rare cases fatalities may occur (reviewed in [131]). Within the natural infection cycle between small mammals and mosquito vectors, humans are considered as dead-end hosts, meaning that the virus is not transmitted from humans to blood-feeding insects and that, consequently, the transmission chain ends in human beings [132]. As LACV was isolated multiple times from *Aedes triseriatus* [133,134,135] this mosquito species, which is native to North America, is considered the main insect vector. Furthermore, LACV has been isolated from the invasive Asian tiger mosquito *Aedes albopictus* [136,137] and the Asian bush mosquito *Aedes japonicus* [138,139]. In experimental infection studies, both proved to be competent vectors of LACV [140,141]. To date, this virus has not been found in midges, suggesting that these do not play an important role in transmission.

Similar to LACV, JCV has a wide geographical distribution throughout North America and causes a mild febrile illness or central nervous system infections inducing meningitis or meningoencephalitis in humans [142]. However, in contrast to LACV, which is associated with clinical disease in children, JCV induces symptoms also in adults. White-tailed deer and other ungulates are considered the primary vertebrate hosts [143]. The first virus isolation succeeded from a pool of *Aedes abserratus* [144]. Since then, JCV has been found in varying but great numbers of different species of Culicidae (*Aedes* spp., *Anopheles* spp., *Culiseta* spp., *Psorophora* spp.) and Tabanidae (*Chrysops* spp., *Hybomitra* spp.) [145]. In the US state of Connecticut, the predominant vector species seems to be *Aedes canadensis*, while in northeastern New York and in Michigan, JCV is predominantly transmitted by *Aedes provocans* [146,147]. *Aedes intrudens*, *Aedes abserratus* and *Aedes punctor* seem to be important vectors in Massachusetts [148], *Aedes stimulans* in northern Indiana [149], and *Aedes vexans* in North Dakota [150].

KEYV is distributed on the east coast of North America from Florida, where the virus was firstly discovered in 1964 from *Aedes atlanticus* in Keystone [151], up to Maryland in the north and Texas in the west. Vertebrate hosts seem to be mammals such as squirrels, raccoons, and white-tailed deer [152,153,154], while the main insect vectors are mosquitoes such as *Aedes atlanticus* [155], various *Aedes* spp. (e.g., *vexans*, *tiseriatus*, *taeniorhynchus*, *infirmatus*, *canadensis*), and some *Culex* spp. [156]. In humans, antibodies against KEYV have been repeatedly detected [157], and the virus itself was isolated from individuals showing clinical symptoms such as fever and rash [158].

TAHV is endemic in Asia and throughout Africa and represents the first mobovirus pathogenic to humans found in Europe [130]. It may induce a febrile illness referred to as Valtice fever, which occurs mainly in children and is characterized by influenza-like symptoms. In rare cases, it may cause meningitis or atypical pneumonia [159]. Vertebrate hosts other than humans are small mammals such as rabbits or hedgehogs [160,161]. Arthropod vectors are most likely mosquitoes of the genera *Aedes*, *Culex*, and *Anopheles*; however, experimental vector competence studies are again missing. Originally, TAHV was isolated from *Aedes vexans* and *Aedes caspius* mosquitoes in the villages Ťahyňa and Križany, Czechoslovakia [162]. Following its initial discovery, TAHV has been isolated from several mosquito species such as *Aedes vexans*, *Aedes cantans*, *Aedes caspius*, *Aedes sticticus*, *Aedes diantaeus*, *Aedes hexodontus*, *Culex pipien*s and *Anopheles hyrcanus* throughout Europe [163,164,165,166,167,168,169].

## 6. Responses to *Culicoides*-borne Arbovirus Incursions

Since treatment options are not available for *Culicoides*-borne arbovirus infections, other possibilities are discussed to prevent clinical disease in vertebrate hosts and virus spread into unaffected regions. Trading and movement restrictions are put in place for livestock and their products (e.g., semen, embryos) to reduce the risk of virus introductions into new areas [22,103]. For *Culicoides*-borne diseases with teratogenic effects such as AKAV, SBV, or SHUV, livestock-management measures could be effective. The mating period of livestock could be adjusted to avoid that naïve females are in the critical phase of pregnancy during the season of the highest biting midge activity. Another possibility is to ensure that dams acquire immunity before they conceive for the first time, which could be achieved by exposing the youngstock to potentially infected vectors. However, this concept requires a high number of infected insect organisms every year and a very high transmission rate of the virus from the vector to the vertebrate host to ensure that every young animal is bitten and infected. Hence, a much more reliable method to acquire immunity is by vaccination [50]. For some of the livestock-affecting simbuviruses including AKAV, AINOV, and SBV, vaccines are commercially available [170,171,172,173], and European experiences during the bluetongue outbreak from 2006 to 2009 demonstrated that vaccination campaigns against a *Culicoides*-borne disease can play a major role in reducing virus circulation or even in eradicating the disease from a given region [174,175]. For the zoonotic OROV, however, there is no vaccine available yet [176].

From the entomological point of view, the application of insecticides or repellents could be taken into consideration to prevent vectors from biting susceptible animals. In addition, these could be housed in insect-proof (e.g., screened) stable buildings. However, most of these measures seem unhandy, expensive, impracticable and have either none or only limited effect [177,178]. For humans with transient exposure, the use of repellents is a suitable measure to protect themselves from insect bites; *N*,*N*-diethyl-m-toluamide (DEET) is considered the gold standard repellent [177,179].

In addition, measures controlling vector development should be considered. These may include environmental interventions to remove larval breeding sites and the control of adult midges by residual insecticide spraying of surfaces where adult biting midges rest within animal stables. However, due to the broad range of habitats and breeding sites used by biting midges, insecticide treatment and removal of breeding sites are of limited success in biting midge control [177].

In conclusion, *Culicoides*-borne diseases are difficult to control by vector management alone, while vaccinating livestock may represents an effective tool for disease prevention or even for eradicating a disease from a region.

## 7. Concluding Remarks

For veterinary medicine and veterinary public health, *Culicoides* biting midges are of great importance as they transmit a multitude of viruses of great impact. In contrast, they only seem to play a minor role for human public health. Due to their potential to contribute to unexpected disease emergence they should be thoroughly surveyed. Multiple detections of novel viruses or introductions of known pathogens into previously unaffected regions during the past decade demonstrate in an impressive fashion that arbovirus outbreaks are hard to predict in space and time [180].

From the genus *Orthobunyavirus*, the emergence of SBV in Europe or the incursion of SHUV into the Middle East are prominent examples [79,118]. To be prepared for putative future outbreaks of arboviral diseases, it is critical to carry out profound research and surveillance on biting midges and their role as vectors.

## Figures and Tables

**Table 1:** Classification and distribution of Simbu serogroup viruses and vector species responsible for their transmission.

Virus Species	Virus (Abbreviation)	First Isolation			Insect Vector		Distribution		
		Year	Country	Organism	Putative Vectors	Demonstrated Vectors	Continent	Animal Hosts (Virus Detection)	Reference (1st Description)
*Akabane orthobunyavirus*	Akabane virus (AKAV)	1959	Japan	mosquitoes	biting midges, mosquitoes	*C. brevitarsis*, *C. variipennis*^1^	Asia, Africa, Australia	ruminants, swine	[58]
	Tinaroo virus (TINV)	1978	Australia	*Culicoides* midges	biting midges (*C. brevitarsis*)		Australia	ruminants	[59]
	Yaba-7 virus (Y7V)	1963	Nigeria	mosquitoes	mosquitoes		Africa		[60]
*Aino orthobunyavirus*	Aino virus (AINOV)	1964	Japan	mosquitoes	mosquitoes, biting midges (*C. brevitarsis*)		Asia, Australia	ruminants	[61]
*Buttonwillow orthobunyavirus*	Buttonwillow virus (BUTV)	1962	USA	Cottontail rabbit	midges (*C. variipennis*)		North America	leproids	[62]
*Cat Que orthobunyavirus*	Cát Quế virus (CQV)	2004	Vietnam	mosquitoes	mosquitoes (*Culex* spp., *Anopheles* spp., *Mansonia spp.*)		Asia	swine, birds	[63]
	Oya virus (OYAV)	1999	Malaysia	swine	mosquitoes		Asia	swine, humans	[64]
*Faceys paddock orthobunyavirus*	Facey’s paddock virus (FPV)	1974	Australia	mosquitoes	mosquitoes (*Culex annulirostris*), midges		Australia		[65]
*Ingwavuma orthobunyavirus*	Ingwavuma virus (INGV)	1959	South Africa	spectacled weaver	mosquitoes (*Culex* spp., *Monsonia* spp.), biting midges		Africa, Asia	swine, dogs, birds	[66]
*Jatobal orthobunyavirus*	Jatobal virus (JASTV)	1985	Brazil	coati	mosquitoes, biting midges		South America	coati	[67]
*Leanyer orthobunyavirus*	Leanyer virus (LEAV)	1974	Australia	mosquitoes	mosquitoes (*Anopheles meraukensis*)		Australia	cattle, wallabies, dogs	[68]
*Manzanilla orthobunyavirus*	Manzanilla virus (MANV)	1954	Trinidad	Howler monkey	mosquitoes (*Culex tritaeniorhynchus*), biting midges		Central America, Asia		[69]
	Inini virus (INIV)	1973	French Guaiana	Aracari bird			South America	birds	[70]
*Mermet orthobunyavirus*	Mermet virus (MERV)	1964	USA	purple martin	mosquitoes (*Culex* spp.)		North America	birds	[71]
*Oropouche orthobunyavirus*	Iquitos virus (IQTV)	1999	Peru	human	midges		South America	humans	[72]
	Madre de Dios virus (MDDV)	2007	Peru	human	mosquitoes, biting midges		South America	humans, monkeys	[73]
	Oropouche virus (OROV)	1955	Trinidad	human	mosquitoes (*Aedes* spp., *Coquillettidia venezuelensis*, *Culex quinquefasciatus*), biting midges	*C. paraensis*	Central/South America	sloths, non-human primates, rodents, birds, humans	[74]
	Perdões virus (PDEV)	2012	Brazil	black-tufted marmoset			South America	non-human primates	[75]
	Pintupo virus (PINTV)		Panama	sloth	biting midges		Central America	sloths	[76]
*Peaton orthobunyavirus*	Peaton virus (PEAV)	1976	Australia	*Culicoides* spp.	biting midges (*C. brevitarsis, C. imicola, C. jacobsoni*)		Australia, Asia	ruminants, horses	[77]
*Sabo orthobunyavirus*	Sabo virus (SABOV)	1966	Nigeria	goat	biting midges		Africa	goat	[60]
*Sango orthobunyavirus*	Sango virus (SANV)	1965	Nigeria	cattle	mosquitoes, biting midges		Africa	cattle	[60]
*Schmallenberg orthobunyavirus*	Douglas virus (DOUV)	1978	Australia	cattle	biting midges (*C. brevitarsis*)		Australia/Oceania	ruminants	[59]
	Sathuperi virus (SATV)	1957	India	mosquitoes	mosquitoes (*Culex vishnui*), biting midges (*C. oxystoma*)		Asia, Africa	ruminants	[78]
	Schmallenberg virus (SBV)	2011	Germany	cattle	biting midges	biting midges (*C. obsoletus, C. scoticus, C. chiopterus, C. imicola, C. sonorensis* ^1^)	Europe	ruminants	[79]
	Shamonda virus (SHAV)	1965	Nigeria	cattle	biting midges		Africa, Asia	ruminants	[60]
*Shuni orthobunyavirus*	Kaikalur virus (KAIV)	1971	India	mosquitoes	mosquitoes		Asia		[80]
	Shuni virus (SHUV)	1966	Nigeria	cattle	mosquitoes (*Culex theileri*), biting midges	biting midges (*C. nubeculosus* ^1^, *C. sonorensis* ^1^)	Africa, Asia	ruminants, horse, (humans?)	[60]
*Simbu orthobunyavirus*	Para virus (PARAV)		Argentina	mosquitoes	mosquitoes		South America		[81]
	Simbu virus (SIMV)	1955	South Africa	mosquitoes	mosquitoes (*Aedes* spp.), biting midges		Africa		[82]
*Thimiri orthobunyavirus*	Thimiri virus (THIV)	1963	India	Indian Pond Heron	biting midges (*C. histrio*)		Asia	birds	[83]
*Utinga orthobunyavirus*	Utinga virus (UTIV)	1965	Brazil	brown-throated sloth	?		South America	sloths	[84]

^1^ laboratory-adapted species, not naturally found in areas where the respective virus circulates.
